# TIMP2 is a Poor Prognostic Factor and Predicts Metastatic Biological Behavior in Gastric Cancer

**DOI:** 10.1038/s41598-018-27897-x

**Published:** 2018-06-25

**Authors:** Wei Wang, Yifan Zhang, Mingxing Liu, Yang Wang, Tao Yang, Dongsheng Li, Feng Ding, Guang Bai, Qing Li

**Affiliations:** 1 Department of General Surgery, The First Affiliated Hospital of Jinzhou Medical University, Jinzhou, Liaoning 121001 China; 2 Department of Internal Medicine, The Third Affiliated Hospital of Jinzhou Medical University, Jinzhou, Liaoning 121001 China

## Abstract

To explore the prognostic related factors and mechanisms of gastric cancer (GC), we performed the systematic analysis with integrated bioinformatics tools based on multiple on-line datasets. With uni-variate COX analysis, we screened out 37 survival hazardous genes in GC. Further GO assays disclosed that the signatures related with extracellular matrix and structure, and the functions of “cell adhesion molecule binding” and “integrin binding” were the vital mechanisms of disease progression, and tissue inhibitor of metalloproteinase-2 (TIMP2) was the potential biomarker for prognosis. Based on GSEA, GSVA and GCN, TIMP2 was demonstrated to interact with multiple integrin pathways and involve in the regulation of EMT, cell adhesion, and angiogenesis of GC. The associations of TIMP2 expression with reduced OS and RFS of patients were declared by Kaplan-Meier analysis, and further confirmed by 1000 internal bootstrap replications and external KM plotter analysis. With multi-variate COX regression and time-dependent ROC analysis, we validated the prediction independency and capacity of TIMP2 for prognosis. The relationships of TIMP2 with clinicopathological characteristics were also uncovered. Taken together, our findings identify TIMP2 as the novel candidate biomarker for poorer outcome of GC patients, and revealed the underlying functions of TIMP2 and the potential mechanisms for GC progression.

## Introduction

Gastric cancer (GC) is one of the most fetal diseases worldwide, and accounts for the fourth most common cancer and the second leading cause of cancer death^1^. High rate of advanced-stage diagnosis, treatment resistance, and later recurrence and metastasis greatly contribute to the poor prognosis of patients^2^. However, neither the traditional extensively used clinicopathological systems nor the novel molecular signatures could well interpret the malignant behavior and characteristics of GC, based on their limited accuracy and availability for the prognosis prediction in patients^3,4^. Therefore, much of the underlying mechanisms for GC development are still unclear, and more prognosis biomarkers of GC are awaiting uncovered.

High throughput genomic studies based on big cohort could present treasurable clues for the discovery of cancer development mechanisms and novel prognostic factors^4^. The data from the Cancer Genome Atlas (TCGA), Gene Expression Omnibus (GEO) and Sequence Read Archive (SRA) involve dozens of cancer types and hundreds of cases for each cancer type, and have become the important study resources based on the great superiority of more variety and objectivity^5^. Moreover, mutual validations with different datasets and computational methods could be performed to reduce the false negative rate of big data assays^6^. Previously, multiple survival assays using online genomic data have been performed in GC^7,8^. However, most studies were more focused on data mining in a single dataset; lack of systematic and overall evaluation of related data; and absence of pre-analysis case selection according to clinicopathological information, thus provided the results with limited applicability and reliability.

In the current study, we systematically evaluated the prognosis related signatures and biomarkers of GC by combining integrated bioinformatics tools and mutual confirmation method based on multiple genomic-profiling datasets from TCGA and GEO. Using the whole mRNA expression profiles from 872 patients, 37 genes were filtered out to be related with worse outcomes of GC patients by COX assays. Based on further genetic ontology (GO) analysis, the extracellular matrix- related genes with the molecular functions of “cell adhesion molecule binding” and “integrin binding” might be vital for patients’ prognosis, and tissue inhibitor of metalloproteinase-2 (TIMP2) were supposed to be the potential key biomarker for GC progression. Moreover, TIMP2 was proved to increase the prediction accuracy of traditional clinicopathological characteristics (CPPs) for GC prognosis. Subsequent Gene Set Enrichment Analysis (GSEA), gene set variation analysis (GSVA) and co-expression network (GCN) assays shed light on the underlying functions of TIMP2 in GC. The results might enrich our knowledge on the tumorigenesis and development of GC, and also present more evidences to clarify the paradoxical role of TIMP2 in cancers.

## Results

### Basic characteristics of patients

The analysis procedure of the current study was shown in Supplementary Fig. 1, and the GC patients with the available data for the sequential analysis were selected (Supplementary Table 1). Among the 300 patients in GSE62554, most have tumors at T2 (62.4%), N1 (43.7%), M0 (91.0%), and TNM III (31.7%) stage. In GSE15459, most of the 192 tumors were intestinal (51.6%) type and stage III (37.5%). Most of the 380 patients in TCGA dataset have tumors with stage T3 (45.5%), N0 (30.8%), M0 (89.7%), and III (43.9%). The deaths occurred in GSE62254, GSE15459, and TCGA cohorts were135 (45%), 95 (49%), and 147(40%), respectively.

### Identification of survival related genes and the key signatures

**Figure 1:**
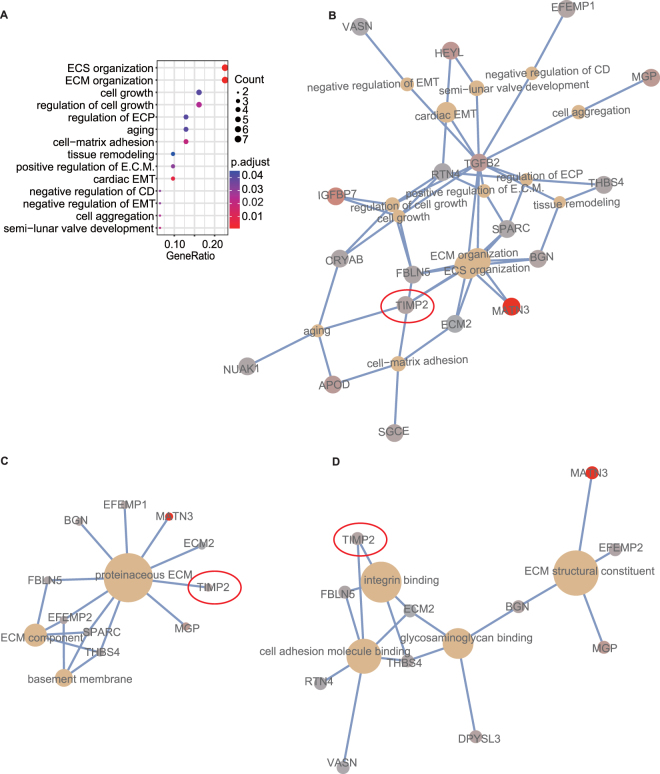
GO enrichment analyses for the 37 prognostic hazardous genes disclose the potential mechanisms of GC progression. **(A)** GO-BP pathways are significantly enriched. Each dot represents a specific GO-BP term, with the count number and the corresponding P value, respectively, indicated by the size and the color of the dot. The genes associated with the enriched GO-BP **(B)**, GO-CC **(C)**, and GO-MF **(D)** terms are illustrated by the cnetplots, with the size of each node representing the overlapped genes in each term. EMT: epithelial to mesenchymal transition, epithelial mesenchymal transition; E.C.M.: epithelial cell migration; ECP: epithelial cell proliferation; CD: cartilage development; ECM: extracellular matrix; ECS: extracellular structure.

According to the uni-variate COX and KM plotter analysis, the 3 datasets shared totally 38 survival-related genes, including 1 protective (hazard ratio (HR) < 1, E2F2) and 37 hazardous (HR > 1) genes (Supplementary Table 2). In the GO analyses using the 37 hazardous genes, totally 14 biological process (BP) terms were significantly enriched, with “extracellular structure (ECS) organization” and “extracellular matrix (ECM) organization” as the most prominent signatures (Fig. 1A and B). In the GO analysis for cellular component (CC), ECM related signatures were found to be enriched (Fig. 1C). Moreover, GO-molecular function (MF) analysis identified “cell adhesion molecule binding” and “integrin binding” as the important functional signatures (Fig. 1D). Several genes, including ECM2, TGFB2, and TIMP2, were consistently found to be associated with the above key signatures in all three GO analyses.

### The relationship of TIMP2 with the integrin family

**Figure 2:**
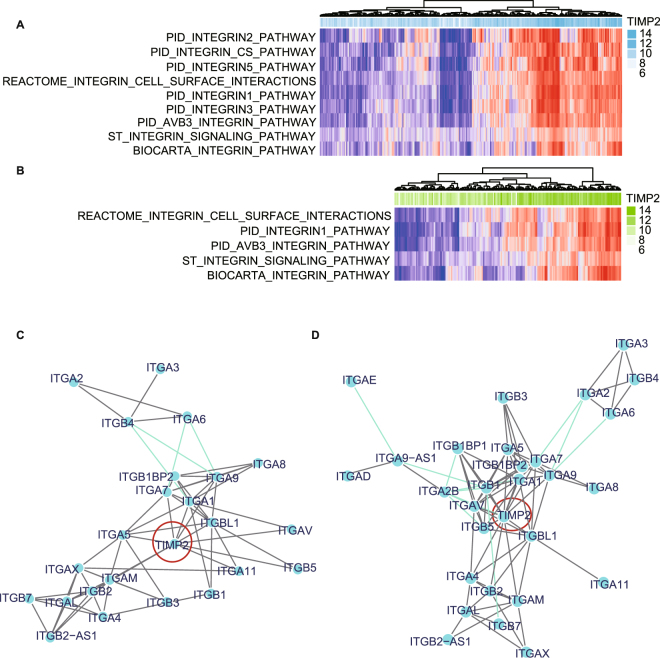
Sequential GSVA and GCN analysis uncover the correlation factors of TIMP2. There are respective 9 **(A)** and 5**(B)** integrin-related signatures enriched by high TIMP2 expression in the GSVA based on GSE62254 and GSE15459. GCN analysis declare 10 **(C)** and 14 **(D)** genes associated with high TIMP2 expression in GSE62254 and GSE15459, respectively.

As integrin family have been reported to be important for ECS and ECM, and TIMP2 were currently found to be related with “integrin binding”, we further explored the relationship of TIMP2 with integrin-related signatures using GSVA, which allows to assess the underlying pathway activity variation via transforming the gene into a signature/gene set by sample matrix without the a priori knowledge of the experimental design. There were respective 9 and 5 integrin pathways associated with TIMP2 expression in GSE62254 (Fig. 2A) and GSE15459 (Fig. 2B). In GCN assays, TIMP2 was shown to respectively co-express with 10 genes in GSE62254 (Fig. 2C), and 14 genes in GSE15459 (Fig. 2D). The overlapped 9 genes were ITGA1, ITGA5, ITGA7, ITGA9, ITGAM, ITGAV, ITGB2, ITGB5, and ITGBL1.

### The potential functions of TIMP2 in cancers

**Figure 3:**
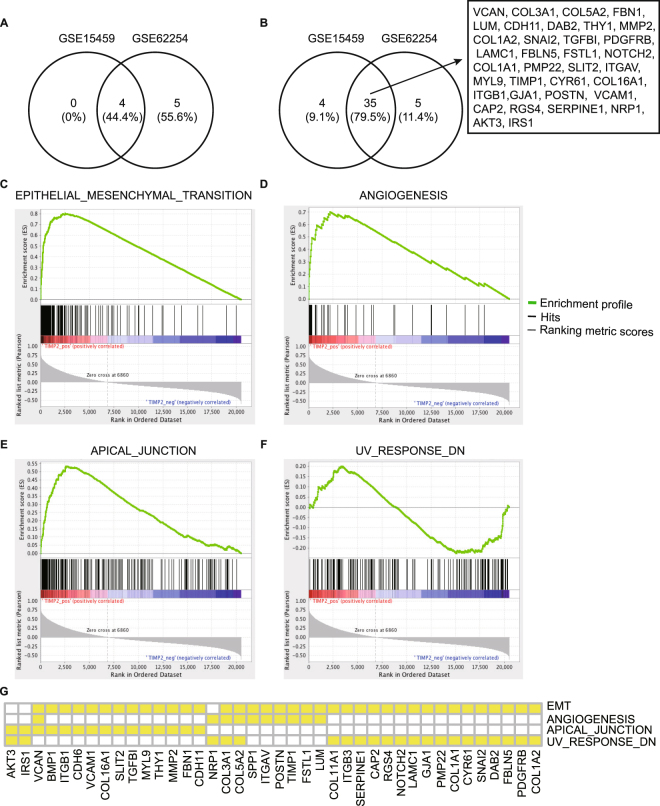
GSEA and LEGA declare the most significant pathways and genes related with TIMP-2 based on GSE62254 and GSE15459. GSE62254 and GSE15459 share 4 gene sets **(A)** and 35 **(B)** leading-edge genes that are related with TIMP-2 expression. The correlation of TIMP2 levels with the 4 hallmark gene-sets, including “EPITHELIAL_MESENCHYMAL_TRANSITION” **(C)**, “ANGIOGENESIS” **(D)**, “APICAL_JUNCTION” **(E)** and “UV_RESPONSE_DN” **(F)**, are illustrated, and the overlapped leading-edge genes in GSE62254 are shown **(G)**.

To further disclose the potential functions of TIMP2 in cancers, we performed GSEA, a robust computational method to identify a-priori defined set of genes in a special phenotype. The most significant gene sets enriched in high TIMP2 phenotype were ordered by significance (false discovery rate (FDR) q- and P-values) and listed in Table 1. As shown in Fig. 3(A,C–F), four hallmark gene-sets, including “ANGIOGENESIS”, “APICAL_JUNCTION”, “EPITHELIAL_MESENCHYMAL_TRANSITION (EMT)” and “UV_RESPONSE_DN”, were shared by the both GSE datasets and supposed to be the vital signatures of high TIMP2 expression. As shown in Fig. 3B and G, GSE62254 and GSE15459 overlapped a number of leading-edge genes, such as cell adhesion molecule related genes including COL3A1, COL5A2, COL1A2, COL1A1, and COL16A1, and integrin family genes including ITGAV and ITGB1.

**Table 1:** Gene set enrichment analysis (GSEA) and leading-edge gene assays according to the levels of TIMP-2 in GSE62254 and GSE15459.

	GSE62254						GSE15459				
	Size	ES	P	FDR.q.	Rank_max_	Leading edge	ES	P	FDR.q.	Rank_max_	Leading edge
ANGIOGENESIS	35	0.70	0.002	0.00	2167	tags = 51%, list = 11%, signal = 57%	0.69	0.004	0.07	3002	tags = 57%, list = 15%, signal = 67%
APICAL_JUNCTION	190	0.53	0.000	0.00	2746	tags = 38%, list = 13%, signal = 44%	0.48	0.002	0.08	3276	tags = 40%, list = 16%, signal = 47%
COAGULATION	135	0.60	0.002	0.00	2821	tags = 40%, list = 14%, signal = 46%	—				
EMT	196	0.80	0.000	0.00	2525	tags = 73%, list = 12%, signal = 83%	0.76	0.000	0.06	2375	tags = 64%, list = 12%, signal = 72%
IR	197	0.63	0.004	0.01	4572	tags = 63%, list = 22%, signal = 81%	—				
KRAS_SIGNALING_UP	193	0.54	0.000	0.01	3309	tags = 45%, list = 16%, signal = 53%	—				
MYOGENESIS	197	0.59	0.000	0.00	3377	tags = 47%, list = 16%, signal = 56%	—				
TGF_BETA_SIGNALING	53	0.55	0.004	0.02	2997	tags = 40%, list = 15%, signal = 46%	—				
UV_RESPONSE_DN	139	0.59	0.002	0.00	3269	tags = 47%, list = 16%, signal = 56%	0.64	0.000	0.08	4192	tags = 61%, list = 20%, signal = 76%

EMT: EPITHELIAL_MESENCHYMAL_TRANSITION; IR: INFLAMMATORY_RESPONSE.

### The survival prediction value of TIMP2 and the relationship with CPPs in GC

**Figure 4:**
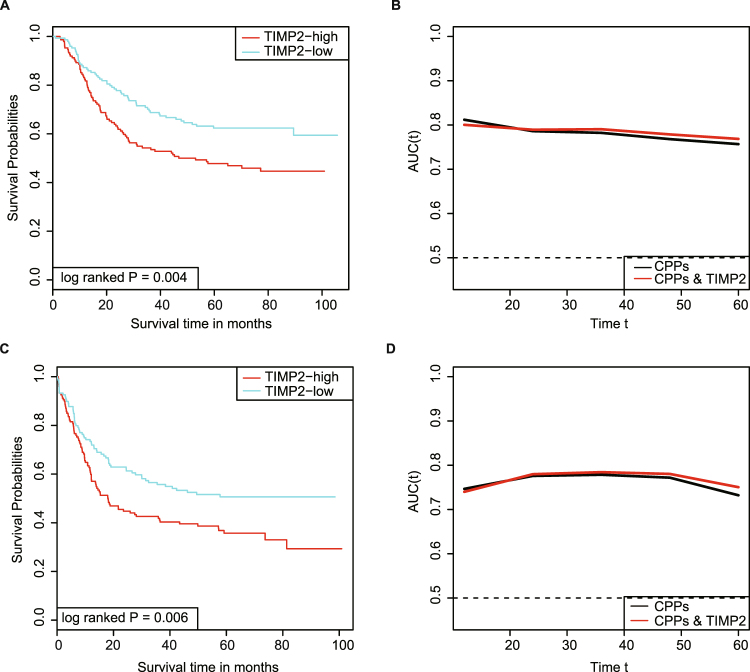
High TIMP2 predicts poorer survival of GC patients in GSE62254. **(A)** K-M analysis identified the prognosis significance of TIMP2 for OS of patients in GC. **(B)** TIMP2 combining with CPPs increases the prediction accuracy of traditional CPPs for OS. **(C)** TIMP2 expression predicts reduced RFS of patients as demonstrated by K-M analysis. **(D)** The addition of TIMP2 to CPPs slightly improves the prediction capacity of CPPs for RFS.

TIMP2 expression was demonstrated to predict shorter overall survival (OS) and recurrence free survival (RFS) of patients in the Kaplan-Meier (K-M) analysis (log-rank P = 0.004 and 0.006, respectively), and was further identified as the independent predictor for OS and RFS in the multi-variate COX analysis, respectively, using GSE62254 (Fig. 4A and C; Table 2) and GSE15459 (Supplementary Fig. 2A). The COX models based on the databases were both successfully validated by 1000 times bootstrapping (Table 2). Moreover, the inclusion of TIMP2 levels to the prognostic model using traditional CPPs slightly improved the prediction ability for OS and RFS (Fig. 4B and D, Supplementary Fig. 2B), as demonstrated by the increase of the resulting area under curve (AUC) values.

**Table 2:** Multivariate COX analysis for the independent predictors of overall survival and recurrence free survival of GC patients in GSE62254.

	OS				RFS			
	HR	95% CI	P - value	Bootstrapping 95% CI	HR	95% CI	P - value	Bootstrapping 95% CI
TIMP2	1.39	1.16–1.84	0.001	1.17–1.86	1.44	1.17–1.78	<0.001	1.18–1.80
Age	1.02	1.01–1.04	0.003	1.01–1.04	1.02	1.01–1.04	0.002	1.01–1.04
T-stage	1.16	1.02–1.68	0.034	1.00–1.71	—	—	—	—
N-stage	1.77	1.59–2.41	<0.001	1.61–2.42	1.82	1.52–2.19	<0.001	1.54–2.20
M-stage	2.01	1.42–3.66	<0.001	1.40–3.72	2.03	1.29–3.21	0.002	1.30–3.27

OS: overall survival; RFS: recurrence free survival.

**Figure 5:**
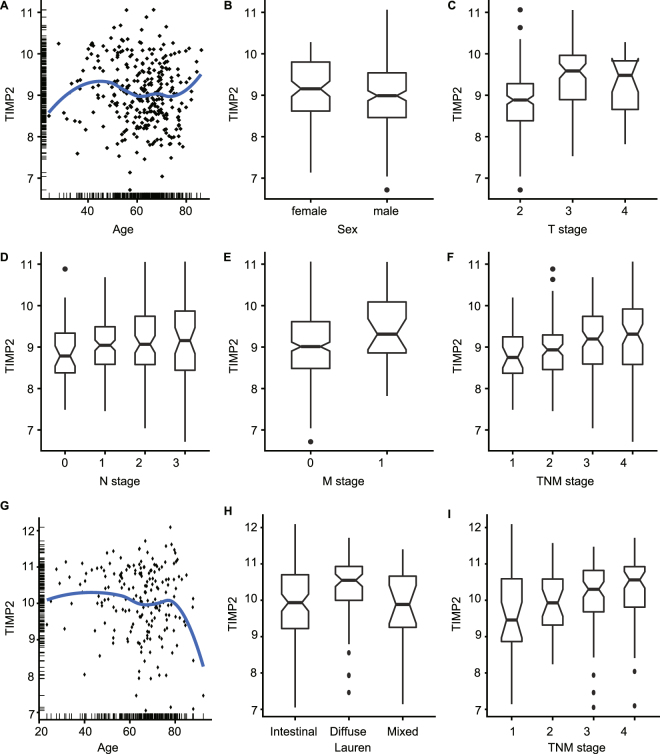
High TIMP2 is related with multiple CPPs involved in GC progression in GSE62254 and GSE15459. TIMP2 expression is significantly correlated with age **(A)**, sex **(B)**, T- **(C)**, M- **(E)**, and TNM-stage **(F)**, and marginal related with N-stage **(D)** in GC cohort of GSE62254. High TIMP2 has no relationship with age **(G)**, whereas statistically correlates with Lauren type **(H)** and TNM-stage **(I)** of cancer in GSE15459.

The associations of TIMP2 with CPPs were shown in Table 3, and Fig. 5. TIMP2 expression was significantly positively correlated with the age (P = 0.047), sex (P = 0.039), T stage (P < 0.001), M stage (P = 0.021), and TNM stage (P < 0.001) of patients in GSE62254, and the Lauren type (P = 0.020) and TNM stage (P = 0.002) of GC in GSE15459.

**Table 3:** The relationship of TIMP2 expression with CPPs of GC patients in GSE62254 and GSE15459.

Variable	GSE62254			GSE15459		
	N (%)	TIMP-2 (median, IQR)	P	N (%)	TIMP-2 (median, IQR)	P
Age (median, IQR)	64 (55–70)	—	0.047	67 (57–73)	—	0.164
Sex			0.039			0.074
Female	101 (33.9)	9.16 (8.62–9.80)		67 (34.9)	10.37 (9.78–10.87)	
Male	197 (66.1)	8.99 (8.46–9.54)		125 (65.1)	10.06 (9.26–10.75)	
Lauren’s			—			0.020
Intestinal	—	—		99 (51.6)	9.93 (9.22–10.70)	
Diffused	—	—		75 (39.1)	10.55 (10.00–10.93)	
Mixed	—	—		18 (9.4)	9.89 (9.25–10.66)	
T stage			<0.001			—
T1	—	—		—	—	
T2	186 (62.4)	8.89 (8.38–9.28)		—	—	
T3	91 (30.5)	9.59 (8.89–9.96)		—	—	
T4	21 (7.0)	9.48 (8.66–9.83)		—	—	
N stage			0.090			—
N0	38 (12.8)	8.79 (8.38–9.34)		—	—	
N1	130 (43.6)	9.04 (8.58–9.49)		—	—	
N2	79 (26.5)	9.07 (8.58–9.74)		—	—	
N3	51 (17.1)	9.16 (8.44–9.87)		—	—	
M stage			0.021			—
M0	271 (90.9)	9.01 (8.48–9.61)		—	—	
M1	27 (9.1)	9.31 (8.86–10.09)		—	—	
TNM stage			<0.001			0.002
I	30 (10.1)	8.75 (8.37–9.25)		31 (16.1)	9.46 (8.87–10.59)	
II	96 (32.2)	8.93 (8.46–9.29)		29 (15.1)	9.93 (9.32–10.58)	
III	95 (31.9)	9.19 (8.59–9.74)		72 (37.5)	10.30 (9.68–10.82)	
IV	77 (25.8)	9.31 (8.58–9.92)		60 (31.2)	10.56 (9.81–10.92)	

IQR, interquartile range.

## Discussion

With comprehensive bioinformatics analysis of 3 big GC datasets, we currently screened out 37 genes that could predict poorer outcome of patients, and further identified that TIMP2 might play a key role in the prognosis of GC through directly binding with integrin and working on cell adhesion, EMT, and angiogenesis of cancers.

TIMP2 is originally found to prohibit cell proliferation and migration *in vitro* via inhibiting the function of MMPs, but latterly disclosed indeed a factor full of puzzling paradox by multiple clinical studies. On one hand, TIMP2 was found to predict better prognosis in endometrial^9^ and pancreatic carcinomas^10^, whereas indicate poorer outcome in neuroblastoma, and head and neck, hepatocellular, Canine mammary, laryngeal, renal, colorectal, oral or tong squamous cell cancers^11–18^. On the other hand, conflicting evidences were present by different studies about the prognostic role of TIMP2 in the same type of cancer, such as breast^19,20^, lung^4,21^, cervical^2,22,23^, ovarian^24–26^, and bladder cancer^27–29^. In GC, TIMP2 was declared to express in cancer, mesenchymal or immune cells, with the clinical role still ambiguous. For example, TIMP2 was shown to predict disease progression^30–32^ in some studies, while demonstrated to have no relationship with CPPs^33^ or survival^30,33,34^ in others.

In the current study, we found that TIMP2 overexpression could predict poorer survival in GC, which is believed to be credible due to the following reasons. First, the clinical role of TIMP2 in GC was explored based on all the 3 biggest available on-line omics datasets of clinical patients, and further confirmed by 1000 internal bootstrap replications and external KM plotter analysis. Second, the independent prediction ability of TIMP2 for patients’ survival was constructed using COX regression model, and further validated by ROC analysis, which demonstrated that the addition of TIMP2 increased the prediction accuracy of the traditional prognostic model using CPPs. Third, the correlations of TIMP2 with Lauren type, and T, M, and TNM stage, were consistent with multiple previous reports, in which TIMP2 was significantly related with the Lauren classification in GC^30^, the distant metastasis in soft tissue sarcoma^35^, as well as gastric^30,34,36^, colorectal^37^, breast^38^, and Canine mammary cancer^14^, the local invasion in invasive prolactinomas^39^, and gastric^32,40^ and thyroid papillary cancer^41^, and the T or TNM stage in oral^42,43^, bladder^29^ and cervical cancer^23^. Besides, our further Oncomine analysis revealed significant higher TIMP2 in GC than non-cancer tissues, which indicates the potential key role of TIMP2 in the oncogenesis and development of GC (data not shown). Moreover, our explorations for the mechanisms of GC progression and TIMP2 functions might present more certifications for the current novel findings.

Consistent with previous reports, our analysis disclosed that TIMP2 involves in the regulation of EMT, cell adhesion, and angiogenesis, and interacts with cell adhesion molecular, and multiple integrin pathways and family members. Given the fact that TIMP2 was found to inhibit metastasis and angiogenesis *in vitro* on one hand, but on the other hand high TIMP2 correlates with malignant phenotype and unfavorable prognosis of cancer patients in lots of clinical assays^44–47^, our current study also encounters with the existing unsolved TIMP2 paradox and serves as another evidence for the paradoxical phenomenon. The potential explanations for the contradictions are as follows based on previous reports. First, the recorded concentrations of TIMP2 in tissues and biological fluids are much lower than the used concentrations *in vitro* experiments^44^. Second, TIMP2 is demonstrated to control cell fates through a proteolytic and a non-proteolytic mechanism^44,48^. In the non-proteolytic manner, TIMP2 was found to induce an intracellular ERK signaling cascade at the physiologically-relevant, low concentrations, and thus further disclosed to result in a burst in cancer cell migration^48^, as well as proliferation^4^. To solve the puzzling TIMP2 paradox, more *in-vivo* and high through-put data studies are expected.

The limitations of the current study are listed as follows. First, the role and the function of TIMP2 are conducted by bioinformatics methods based on on-line databases and need to be further validated by *in-vitro* and *in-vivo* experimental studies. Second, the ratios of MMPs/TIMP2 were considered meaningful markers for cancer studies but could not be evaluated currently^49,50^. Third, our current study did not evaluate the prognostic role of TIMP2 on the following respects, such as its genetic variations like SNP and methylation, the expression locations including cancer and mesenchymal cells, and the detection samples like tissues and serum^31,37,51,52^. Further studies are warranted for such topics.

In summary, we firstly uncover the significant prognostic role and the potential function of TIMP2 in GC with multiple datasets and integrated bioinformatics analysis-methods. TIMP2 is identified as a potential predictor for poorer outcome of patients, with the predictive accuracy and independency well demonstrated by both external and internal validations. Further GSEA, GSVA and GCN analysis declared the potential mechanisms for the progression of GC, such as cell adhesion; integrin networks, angiogenesis, and so on. Our findings present a novel candidate biomarker for patients’ prognosis in GC, shed lights on the underlying function of TIMP2, and indicate novel interesting topics for future studies.

## Materials and Methods

### Data preprocessing and summarization

The GC online datasets from Gene Expression Omnibus database (GEO, http://www.ncbi.nlm.nih.gov/geo/) were filtered according to the following criteria: 1) include more than 100 cases; 2) provide both clinicopathological and survival information (OS, and/or RFS); 3) use the universal high-throughput platform, such as affymetrix HGU133 plus2 (GPL570) or Illumina HiSeq 2000 instrument (GPL11154). The search strategy was as follows: (gastric cancer) AND ((GPL570) OR GPL11154) Filters: Series; DataSets, and totally 12 datasets were filtered out. After manual screening, two data sets including GSE62254 and GSE15459, were used in the current study. The raw data were preprocessed with Robust Multichip Average (RMA) methods in the default settings. The probe-level data were converted into the corresponding genetic symbols based on the annotation platform. As for a gene corresponding to a plurality of probes, average value was taken as the initial expression value. In addition, the TCGA RNASEQV2 data (level 3) of GC was downloaded from UCSC xena website (http://xena.ucsc.edu/), and cases with primary GC, no history of neo-adjuvant therapy, complete information of survival (OS and/or RFS) and mRNA profile, were included.

GSE62254 and GSE15459 were both performed on platform GPL570 and contained 300 cases from Korea and 192 cases from Singapore, respectively. Totally 20502 genes of each GEO dataset were analyzed for the relationship with the corresponding clinicopathological data, such as gender, age at diagnosis, histological subtypes, TNM stage, OS and/or RFS. According to TCGA, 380 out of 580 samples were selected currently. A total of 20564 genes were taken for the analysis. For the cohort in GSE62254, GSE15459 and TCGA, the median follow-up time, as calculated by the reverse KM method^53^, was respective 76.93, 74.33 and 23.07 months, and the median OS was respective 31.20 months, not reached, and 29.37 months. The CPPs of the 3 datasets were summarized in Supplementary Table 1.

### Uni-variate COX, KM plotter and GO analysis

With each of the three selected datasets, including GSE62254, GSE15459, and TCGA, the prognostic value of each gene was calculated in the uni-variate COX analysis, and the genes with both P < 0.05 and beta_OS_ * beta_RFS_ > 0 were kept for further analysis. Genes were ranked based on the P values in the order from small to large, and the rank number based on P_OS_ and P_RFS_ was, respectively, defined as rank_OS_ and rank_RFS._ For each gene, the overall mean of rank_OS_ and rank_RFS_ was calculated and defined as rank_ave_. The genes of the 3 datasets were, respectively, sorted based on the rank_ave_ in the order from small to large, and the top 1000 genes of each dataset were considered as significant. Then the gene with hazard ratio (HR) > 1 across the 3 datasets was considered survival hazardous, and the gene with HR < 1 across the 3 datasets was considered survival protective. The prognostic significance of the selected genes in GC were further confirmed using KM plotter. With the Bioconductor “clusterProfiler” package, GO-BP, -CC, and -MF were analyzed with the hazardous genes, using P- and FDR q-value < 0.05 as the standard for statistical significance.

### GSVA and GCN construction

To infer specific activated pathways related with TIMP2 expression, we performed GSVA using the c2 curated signatures downloaded from the Molecular Signatures Database (MSigDB). The signatures whose name including “integrin” were chosen and applied to the correlation tests for the relationship between TIMP2 levels and the activation scores of the integrin-related signatures in GSE62254 and GSE15459, respectively. The signatures with Pearson correlation coefficients (PCC) higher than 0.6 were considered significant.

With R/igraph package and cytoscape V3.5.1, GCN was built on the correlation matrix of integrin family genes with TIMP2 in GSE62254 and GSE15459, respectively. PCC for every pair of genes were calculated, and the gene pairs with |PCC| > 0.4 and FDR q < 0.05 were retained.

### GSEA, venn diagram and Leading-Edge Gene Analysis (LEGA)

For GSEA, 50 available hallmark gene sets from MSigDB were used, and the expression levels of TIMP2 were set to annotate the phenotypes of samples. After performing 1,000 permutations, the first 20 signatures with FDR q < 0.1 and P < 0.005 were considered to be significantly enriched. The overlapped enriched hallmark signatures in GSE62254 and GSE15459 were illustrated by venn diagram, and further applied to LEGA.

### Correlation tests, K-M log-rank test, multi-variate COX analysis with bootstrapping, and ROC curve

Based on GSE62254 and GSE15459, spearman correlation tests were, respectively, performed to analyze the correlation of TIMP2 and CPPs, including age, sex, Lauren type, T-stage, N-stage, M-stage, and TNM stage. In the survival assays using K-M method and log-rank test, expression level of TIMP2 was dichotomized as high and low with median as the cutoff value. Multi-variate Cox PH models were used to estimate HR and 95% confidence interval (CI). The final multivariate model was constructed based on the Akaike information criterion (AIC) value using both “backward” and “forward” stepwise selection methods, and further internally validated by coefficients and 95% CIs using bootstrapping (1000 replications). Based on the risk scores, the AUC (t) curve was plotted to illustrate time dependent sensitivity and specificity of corresponding time-dependent receiver-operating characteristics (ROC) at each observed event time.

## Electronic supplementary material

Supplementary file
