# Supplementary material for: TIMP2 is a Poor Prognostic Factor and Predicts Metastatic Biological Behavior in Gastric Cancer
Source: Sci Rep. 2018 Jun 25;8:9629. doi: 10.1038/s41598-018-27897-x (PMC6018464; doi:10.1038/s41598-018-27897-x)
Supplement: Supplementary file 1 — Supplementary file [file 41598_2018_27897_MOESM1_ESM.docx]

**TIMP2 is a Poor Prognostic Factor and Predicts Metastatic Biological Behavior in Gastric Cancer**

**Wei Wang, YiFan Zhang, Mingxing Liu, Yang Wang, Tao Yang, Dongsheng, Feng Ding, Guang Bai, Qing Li**

**Supplementary Table 1.** Basic characteristics of patients in TCGA, GSE15459 and GSE 62254 cohort

|  | TCGA (%) | GSE15459 (%) | GSE62254 (%) |
| --- | --- | --- | --- |
| Age: Median (IQR) | 67 (30 - 90) | 66.6 (23.4 - 92.4) | 46.0 (24.0 - 86.0) |
| Sex |  |  |  |
| Female | 131 (34.5) | 67 (34.9) | 101 (33.7) |
| Male | 249 (65.5) | 125 (65.1) | 199 (66.3) |
| Lauren’s |  |  |  |
| Intestinal | -- | 99 (51.6) | -- |
| Diffuse | -- | 75 (39.1) | -- |
| Mixed | -- | 18 (9.3) | -- |
| T stage |  |  |  |
| T1 | 21 (5.5) | -- | -- |
| T2 | 77 (20.3) | -- | 186 (62.4) |
| T3 | 173 (45.5) | -- | 91 (30.5) |
| T4 | 106 (27.9) | -- | 21 (7.1) |
| X | 3 (0.8) | -- | 2 (0.6) |
| N stage |  |  |  |
| N0 | 117 (30.8) | -- | 38 (12.7) |
| N1 | 101 (26.6) | -- | 131 (43.7) |
| N2 | 75 (19.7) | -- | 80 (26.7) |
| N3 | 77 (20.3) | -- | 51 (17.0) |
| X | 10 (2.6) | -- | -- |
| M stage |  |  |  |
| M0 | 341 (89.7) | -- | 273 (91.0) |
| M1 | 20 (5.3) | -- | 27 (9.0) |
| X | 19 (5.0) | -- | -- |
| TNM stage |  |  |  |
| I | 52 (13.7) | 31 (16.1) | 30 (10.0) |
| II | 119 (31.3) | 29 (15.1) | 96 (32.0) |
| III | 167 (43.9) | 72(37.5) | 95 (31.7) |
| IV | 30 (7.9) | 60(31.3) | 77 (25.7) |
| X | 12 (3.1) | -- | 2 (0.6) |

**Supplementary Table 2.** The correlation of the 37 genes with patients’ survival in GC based on TCGA, GSE62254 and GSE15459 databases using sequential uni- variate COX and KM plotter analysis.

|  | KM plotter-OS | | TCGA-OS | | TCGA-RFS | | GSE62254-OS | | GSE62254-RFS | | GSE15459-OS | |
| --- | --- | --- | --- | --- | --- | --- | --- | --- | --- | --- | --- | --- |
| Gene | HR (95% CI) | P-value | HR (95% CI) | P-value | HR (95% CI) | P-value | HR (95% CI) | P-value | HR (95% CI) | P-value | HR (95% CI) | P-value |
| MATN3 | 1.68 (1.40 - 2.01) | 8.70E-09 | 1.37 (1.18 - 1.58) | 3.08E-05 | 1.60 (1.29 - 1.97) | 1.51E-05 | 1.43 (1.25 - 1.65) | 4.74E-07 | 1.53 (1.34 - 1.76) | 1.25E-09 | 1.39 (1.19 - 1.61) | 1.85E-05 |
| IGFBP7 | 1.47 (1.23 - 1.76) | 2.30E-05 | 1.31 (1.12 - 1.53) | 7.57E-04 | 1.40 (1.11 - 1.76) | 4.44E-03 | 2.01 (1.56 - 2.59) | 6.68E-08 | 1.75 (1.38 - 2.20) | 2.65E-06 | 2.06 (1.40 - 3.01) | 2.22E-04 |
| AKAP12 | 2.22 (1.75 - 2.82) | 1.30E-11 | 1.23 (1.09 - 1.38) | 4.99E-04 | 1.28 (1.08 - 1.51) | 3.73E-03 | 1.46 (1.27 - 1.68) | 7.13E-08 | 1.42 (1.25 - 1.62) | 1.34E-07 | 1.33 (1.12 - 1.57) | 8.87E-04 |
| NREP | 1.61 (1.25 - 2.08) | 2.50E-04 | 1.38 (1.14 - 1.66) | 8.16E-04 | 1.58 (1.18 - 2.13) | 2.48E-03 | 2.05 (1.60 - 2.62) | 1.71E-08 | 1.81 (1.43 - 2.28) | 5.45E-07 | 1.73 (1.22 - 2.46) | 1.95E-03 |
| GLIS2 | 1.99 (1.59 - 2.50) | 7.70E-10 | 1.30 (1.09 - 1.55) | 4.22E-03 | 1.66 (1.24 - 2.23) | 6.11E-04 | 1.53 (1.27 - 1.86) | 1.21E-05 | 1.45 (1.21 - 1.72) | 3.55E-05 | 1.80 (1.39 - 2.33) | 9.96E-06 |
| HEYL | 2.31 (1.84 - 2.91) | 2.20E-13 | 1.31 (1.13 - 1.52) | 2.82E-04 | 1.26 (1.01 - 1.56) | 4.03E-02 | 3.21 (2.26 - 4.56) | 7.38E-11 | 2.75 (1.99 - 3.82) | 1.29E-09 | 2.43 (1.58 - 3.74) | 5.61E-05 |
| TGFB2 | 1.62 (1.35 - 1.94) | 1.90E-07 | 1.47 (1.17 - 1.84) | 8.13E-04 | 1.66 (1.19 - 2.30) | 2.51E-03 | 2.07 (1.54 - 2.77) | 1.23E-06 | 1.85 (1.41 - 2.44) | 9.69E-06 | 1.68 (1.22 - 2.32) | 1.38E-03 |
| MGP | 1.50 (1.25 - 1.80) | 1.40E-05 | 1.14 (1.05 - 1.24) | 2.40E-03 | 1.19 (1.05 - 1.35) | 5.70E-03 | 1.38 (1.23 - 1.55) | 2.68E-08 | 1.28 (1.15 - 1.42) | 4.15E-06 | 1.22 (1.08 - 1.39) | 1.75E-03 |
| VAT1 | 1.85 (1.55 - 2.20) | 3.60E-12 | 1.41 (1.13 - 1.75) | 2.00E-03 | 1.72 (1.24 - 2.38) | 1.12E-03 | 2.03 (1.47 - 2.80) | 1.67E-05 | 1.90 (1.42 - 2.55) | 1.58E-05 | 2.16 (1.40 - 3.33) | 5.21E-04 |
| APOD | 1.96 (1.60 - 2.40) | 4.10E-11 | 1.17 (1.09 - 1.26) | 1.58E-05 | 1.22 (1.09 - 1.35) | 3.01E-04 | 1.24 (1.13 - 1.36) | 2.99E-06 | 1.21 (1.11 - 1.32) | 7.08E-06 | 1.19 (1.06 - 1.32) | 2.07E-03 |
| THSD7B | 2.07 (1.66 - 2.58) | 3.50E-11 | 1.59 (1.13 - 2.22) | 7.02E-03 | 1.82 (1.12 - 2.97) | 1.60E-02 | 1.40 (1.21 - 1.60) | 2.61E-06 | 1.37 (1.19 - 1.57) | 1.36E-05 | 1.77 (1.40 - 2.25) | 2.38E-06 |
| RBMS1 | 1.86 (1.50 - 2.31) | 1.00E-08 | 1.44 (1.16 - 1.79) | 1.01E-03 | 1.54 (1.11 - 2.14) | 1.01E-02 | 1.68 (1.32 - 2.14) | 2.36E-05 | 1.60 (1.28 - 1.99) | 3.04E-05 | 1.51 (1.19 - 1.93) | 8.48E-04 |
| NALCN | 2.24 (1.79 - 2.80) | 4.90E-13 | 1.77 (1.15 - 2.71) | 8.90E-03 | 2.13 (1.21 - 3.75) | 9.15E-03 | 3.37 (1.98 - 5.75) | 7.99E-06 | 2.62 (1.56 - 4.39) | 2.62E-04 | 4.81 (2.62 - 8.81) | 3.69E-07 |
| DPYSL3 | 1.85 (1.51 - 2.27) | 1.20E-09 | 1.16 (1.05 - 1.28) | 2.49E-03 | 1.18 (1.02 - 1.36) | 2.33E-02 | 1.60 (1.34 - 1.92) | 2.33E-07 | 1.47 (1.25 - 1.73) | 4.80E-06 | 1.34 (1.11 - 1.61) | 2.10E-03 |
| TIMP2 | 1.89 (1.52 - 2.35) | 4.90E-09 | 1.28 (1.09 - 1.50) | 2.44E-03 | 1.33 (1.05 - 1.69) | 1.62E-02 | 1.61 (1.29 - 2.03) | 3.43E-05 | 1.50 (1.22 - 1.84) | 1.32E-04 | 1.71 (1.34 - 2.20) | 2.41E-05 |
| SGCE | 1.54 (1.25 - 1.90) | 5.60E-05 | 1.29 (1.11 - 1.50) | 7.32E-04 | 1.32 (1.06 - 1.64) | 1.30E-02 | 1.45 (1.27 - 1.66) | 4.23E-08 | 1.40 (1.24 - 1.58) | 5.12E-08 | 1.24 (1.06 - 1.44) | 6.14E-03 |
| BGN | 1.90 (1.56 - 2.32) | 1.30E-10 | 1.21 (1.06 - 1.38) | 3.65E-03 | 1.29 (1.07 - 1.56) | 8.92E-03 | 1.54 (1.24 - 1.90) | 7.00E-05 | 1.43 (1.18 - 1.73) | 2.97E-04 | 1.67 (1.37 - 2.03) | 3.90E-07 |
| EFEMP1 | 1.41 (1.19 - 1.67) | 9.20E-05 | 1.22 (1.10 - 1.36) | 1.58E-04 | 1.19 (1.03 - 1.39) | 2.10E-02 | 1.46 (1.24 - 1.72) | 5.41E-06 | 1.34 (1.16 - 1.55) | 1.02E-04 | 1.34 (1.12 - 1.62) | 1.75E-03 |
| EFEMP2 | 1.86 (1.56 - 2.23) | 3.20E-12 | 1.25 (1.07 - 1.46) | 5.96E-03 | 1.34 (1.06 - 1.70) | 1.36E-02 | 1.67 (1.39 - 2.02) | 7.39E-08 | 1.57 (1.32 - 1.86) | 2.92E-07 | 1.36 (1.09 - 1.69) | 5.55E-03 |
| FAM19A5 | 2.33 (1.87 - 2.89) | 4.80E-15 | 1.25 (1.05 - 1.50) | 1.42E-02 | 1.37 (1.05 - 1.78) | 2.05E-02 | 1.66 (1.35 - 2.05) | 1.45E-06 | 1.53 (1.25 - 1.87) | 4.50E-05 | 1.52 (1.21 - 1.90) | 2.71E-04 |
| RASSF8 | 1.77 (1.43 - 2.20) | 1.30E-07 | 1.35 (1.15 - 1.58) | 2.00E-04 | 1.32 (1.04 - 1.66) | 2.06E-02 | 3.02 (1.98 - 4.60) | 2.81E-07 | 2.52 (1.71 - 3.72) | 3.05E-06 | 1.51 (1.13 - 2.03) | 5.52E-03 |
| GXYLT2 | 2.46 (1.90 - 3.20) | 3.10E-12 | 1.24 (1.08 - 1.42) | 2.46E-03 | 1.23 (1.00 - 1.50) | 4.48E-02 | 1.46 (1.24 - 1.71) | 4.43E-06 | 1.38 (1.19 - 1.60) | 2.23E-05 | 1.32 (1.14 - 1.52) | 1.80E-04 |
| CCDC92 | 2.17 (1.75 - 2.69) | 2.90E-13 | 1.42 (1.08 - 1.89) | 1.34E-02 | 1.78 (1.15 - 2.75) | 9.38E-03 | 2.39 (1.72 - 3.33) | 2.36E-07 | 2.27 (1.69 - 3.05) | 5.66E-08 | 1.60 (1.15 - 2.23) | 5.19E-03 |
| PCDHB5 | 1.80 (1.45 - 2.24) | 7.20E-08 | 1.35 (1.17 - 1.56) | 3.88E-05 | 1.36 (1.09 - 1.70) | 6.02E-03 | 1.28 (1.15 - 1.43) | 8.99E-06 | 1.28 (1.15 - 1.43) | 4.89E-06 | 1.29 (1.08 - 1.54) | 5.82E-03 |
| CRYAB | 2.48 (1.97 - 3.12) | 8.90E-16 | 1.14 (1.04 - 1.26) | 5.63E-03 | 1.16 (1.01 - 1.32) | 3.84E-02 | 1.40 (1.25 - 1.56) | 1.83E-09 | 1.32 (1.19 - 1.46) | 8.68E-08 | 1.20 (1.06 - 1.36) | 3.51E-03 |
| LHFP | 1.44 (1.21 - 1.71) | 4.40E-05 | 1.25 (1.08 - 1.44) | 2.84E-03 | 1.26 (1.02 - 1.56) | 2.99E-02 | 1.62 (1.37 - 1.92) | 1.62E-08 | 1.51 (1.29 - 1.76) | 2.30E-07 | 1.30 (1.08 - 1.56) | 5.44E-03 |
| FBLN5 | 1.85 (1.50 - 2.29) | 6.60E-09 | 1.25 (1.09 - 1.44) | 1.28E-03 | 1.31 (1.08 - 1.60) | 6.68E-03 | 1.44 (1.23 - 1.68) | 4.69E-06 | 1.39 (1.20 - 1.60) | 8.10E-06 | 1.25 (1.06 - 1.46) | 6.93E-03 |
| THBS4 | 1.57 (1.33 - 1.86) | 1.30E-07 | 1.08 (1.01 - 1.16) | 2.34E-02 | 1.10 (1.00 - 1.21) | 4.15E-02 | 1.24 (1.14 - 1.36) | 2.28E-06 | 1.19 (1.10 - 1.30) | 4.60E-05 | 1.25 (1.13 - 1.39) | 1.13E-05 |
| NUAK1 | 1.74 (1.47 - 2.06) | 9.00E-11 | 1.37 (1.07 - 1.76) | 1.40E-02 | 1.65 (1.13 - 2.41) | 1.02E-02 | 1.43 (1.18 - 1.74) | 3.02E-04 | 1.43 (1.20 - 1.71) | 6.15E-05 | 1.65 (1.30 - 2.08) | 2.82E-05 |
| PDLIM3 | 2.02 (1.60 - 2.54) | 9.80E-10 | 1.14 (1.03 - 1.25) | 8.57E-03 | 1.18 (1.02 - 1.35) | 2.21E-02 | 1.52 (1.30 - 1.78) | 1.25E-07 | 1.42 (1.23 - 1.64) | 2.42E-06 | 1.25 (1.07 - 1.47) | 5.36E-03 |
| SPARC | 1.28 (1.06 - 1.54) | 9.00E-03 | 1.27 (1.09 - 1.47) | 2.04E-03 | 1.26 (1.01 - 1.58) | 4.00E-02 | 1.52 (1.25 - 1.86) | 3.59E-05 | 1.44 (1.2 - 1.73) | 9.56E-05 | 1.46 (1.18 - 1.81) | 5.62E-04 |
| TAGLN | 2.59 (2.04 - 3.29) | 4.40E-16 | 1.11 (1.03 - 1.21) | 9.14E-03 | 1.13 (1.00 - 1.26) | 4.31E-02 | 1.44 (1.28 - 1.62) | 3.20E-09 | 1.37 (1.22 - 1.53) | 4.07E-08 | 1.23 (1.06 - 1.41) | 4.81E-03 |
| RTN4 | 1.37 (1.15 - 1.63) | 3.20E-04 | 1.35 (1.01 - 1.81) | 4.43E-02 | 1.63 (1.06 - 2.51) | 2.67E-02 | 2.97 (1.88 - 4.70) | 3.10E-06 | 2.56 (1.68 - 3.91) | 1.29E-05 | 2.21 (1.39 - 3.53) | 8.76E-04 |
| INMT | 1.98 (1.59 - 2.45) | 3.20E-10 | 1.20 (1.05 - 1.37) | 7.36E-03 | 1.22 (1.01 - 1.48) | 3.90E-02 | 1.41 (1.22 - 1.63) | 4.41E-06 | 1.30 (1.13 - 1.50) | 2.67E-04 | 1.34 (1.12 - 1.61) | 1.32E-03 |
| VASN | 1.92 (1.54 - 2.39) | 3.20E-09 | 1.26 (1.04 - 1.52) | 1.75E-02 | 1.48 (1.11 - 1.97) | 7.38E-03 | 1.59 (1.24 - 2.03) | 2.47E-04 | 1.58 (1.26 - 1.97) | 7.04E-05 | 1.63 (1.20 - 2.21) | 1.91E-03 |
| ZFPM2 | 1.43 (1.21 - 1.70) | 3.40E-05 | 1.38 (1.14 - 1.68) | 1.07E-03 | 1.38 (1.03 - 1.85) | 2.91E-02 | 1.28 (1.15 - 1.43) | 5.27E-06 | 1.23 (1.11 - 1.36) | 7.47E-05 | 1.18 (1.05 - 1.34) | 7.90E-03 |
| ECM2 | 1.22 (1.02 - 1.47) | 2.90E-02 | 1.39 (1.13 - 1.72) | 1.81E-03 | 1.43 (1.04 - 1.96) | 2.66E-02 | 1.59 (1.26 – 2.00) | 9.11E-05 | 1.48 (1.20 - 1.83) | 3.08E-04 | 1.42 (1.11 - 1.81) | 5.15E-03 |

**Supplementary Figure 1. Analysis flow chart illustrates the exploration procedure for the GC prognostic genes and the related mechanisms.**

**Supplementary Figure 2. High TIMP2 predicts poorer survival of GC patients in GSE15459.** **(A)** K-M analysis identified the prognosis significance of TIMP2 for OS of patients in GC. **(B)** TIMP2 combining with CPPs increases the prediction accuracy of traditional CPPs for OS.
